# Building and validating a stigma prediction model for overweight and obese patients with polycystic ovary syndrome (PCOS): A observational study

**DOI:** 10.1097/MD.0000000000048655

**Published:** 2026-05-01

**Authors:** Yahui Bian, Yanan Bian, Zijie Fu, Bianling Xu, Tingting Zhao, Jingjing Wang, Qingya Ma, Xiaodong Li

**Affiliations:** a Department of Gynecology, The First Hospital of Hebei Medical University, Shijiazhuang, China; b Supplies Department, The First Hospital of Hebei Medical University, Shijiazhuang, China; c Reproductive Center, The First Hospital of Hebei Medical University, Shijiazhuang, China.

**Keywords:** clinical prediction models, obese, overweight, polycystic ovary syndrome, stigma

## Abstract

This observational study was designed to establish and validate a stigma prediction model for patients with polycystic ovary syndrome (PCOS). The stigma risk scoring table for overweight and obese patients with PCOS has good predictive ability. When an overweight or obese patient with PCOS presents, the prediction model allows clinic staff to rapidly grade hirsutism, acne, and acanthosis, determine fertility desire, and quantify anxiety. Low-risk patients then receive standard care, whereas high-risk patients receive precision interventions. Unlike the traditional approach, this clinical prediction model incorporates not only laboratory values but also body-image concerns and psychological well-being, providing more comprehensive management for women with PCOS. To preliminarily explore the associations among stigma, PCOS signs, anxiety, and depression. A total of 124 overweight and obese patients with PCOS were selected using convenience sampling. The patients in the order of clinic visit time were divided into a modeling set and a validation set at a ratio of 3:1. Univariate analysis was first performed, normally distributed continuous variables were compared using t-tests, non-normally distributed continuous variables with nonparametric rank-sum tests, and categorical variables with χ^2^ tests. Independent risk factors for stigma were identified using multivariable logistic regression, and a nomogram was constructed. The model’s discrimination and calibration were evaluated with the receiver operating characteristic curve and calibration curve. Internal validation was subsequently conducted on the validation data set to assess model performance comprehensively. Hirsutism (odds ratio [OR]=0.075, 95%Cl: 0.015–0.368) , acne (OR=0.210, 95%Cl: 0.050–0.878) , acanthosis nigricans (OR=0.184, 95%Cl: 0.044–0.073) , fertility requirements (OR=0.212, 95%Cl: 0.051–0.890) , and anxiety (OR=1.217, 95%Cl: 1.074–1.378) were independent influencing factors for stigma in these patients (*P* < .05). The constructed prediction model also demonstrated good predictive ability, with area under the curve values of 0.941 and 0.803 for the modeling and validation sets, respectively. Internal validation using 1000 bootstrap resamples revealed a mean area under the receiver operating characteristic curve area under the curve of 0.941.

## 1. Introduction

Polycystic ovary syndrome (PCOS) is the most common gynecological endocrine disorder in women of childbearing age. According to the criteria of the National Institutes of Health, the prevalence of PCOS ranges from 6% to 10%. In contrast, according to the broader Rotterdam criteria, the prevalence of PCOS can be as high as 15%.^[1]^ Notably, unhealthy dietary habits, stressful lifestyles, and genetic factors are contributing to the development of PCOS.^[2,3]^ Additionally, hyperandrogenism and insulin resistance are the 2 primary pathophysiological cores of PCOS, mutually influencing and causally related to each other^[4]^). Hyperandrogenism leads to clinical manifestations such as hirsutism, acne, and ovulation disorders.^[5]^ Consequently, weight issues and obesity in patients with PCOS deserve our attention, as research shows a correlation between PCOS and overweight/obesity.^[6]^ As such, approximately 40%-70% of patients with PCOS are overweight or obese, with central obesity being predominant.^[7]^ Insulin resistance is also significantly higher in overweight and obese individuals with PCOS compared to those with normal weight.^[6]^ Acanthosis nigricans, a skin condition associated with severe insulin resistance, often presents as velvety hyperkeratosis and gray-brown pigmentation on the skin behind the neck, in the groin area, under the arms, and beneath the breasts^[8]^ Unfortunately, a series of issues such as hirsutism, acne, overweight or obesity, and acanthosis nigricans severely impact the physical health and personal image of patients,^[9]^ leading to psychological stress and feelings of stigma.^[10]^ Thus, this study aims to establish and validate a stigma prediction model for overweight and obese patients with PCOS, providing a basis for the early identification of stigma in patients with PCOS.

## 2. Methods

### 2.1. Study subjects

Using convenience sampling, 124 overweight or obese patients with PCOS who attended the Department of Gynecology at the First Hospital of Hebei Medical University between June 2022 and February 2024 were enrolled. According to the calculation formula for a sample size of 10 events per variable in the clinical prediction model, with 9 predictive variables in this study, the minimum sample size should be 90.^[11]^ Inclusion criteria: Participants who meet the Rotterdam criteria for the diagnosis of polycystic ovary syndrome; BMI ≥ 24.0kg/m^2^ and women aged 18 years and above of childbearing age; no use of corticosteroids within the past 3 months. Exclusion criteria: Presence of severe organic lesions in the reproductive organs; severe organ diseases affecting the heart, liver, kidneys, etc; severe mental disorders; severe endocrine disorders such as thyroid disease and diabetes; psychiatric or endocrine comorbidities.

### 2.2. Research Tools

#### 2.2.1. Assessment of hirsutism, acne, and other physical signs:

Criteria used for assessment are based on the 2023 version of the consensus guidelines for the diagnosis and treatment pathway of polycystic ovary syndrome.^[12]^

#### 2.2.2. Basic Information Questionnaire:

Collect information such as age, height, weight, fertility requirements, and disease-related knowledge.

#### 2.2.3. Anxiety Self-Rating Scale:

Utilized the anxiety self-rating scale created by Zung et al.^[13]^ consisting of 20 items rated on a 4-point scale. Based on the Chinese normative results, the standard score was 1.25 times the total raw score, with a score exceeding 50 indicating the presence of anxiety symptoms. Notably, higher scores indicate more severe anxiety symptoms; Cronbach α was 0.82.^[14]^

#### 2.2.4. Depression Self-Rating Scale:

Utilized the depression self-rating scale created by Zung et al.^[13]^ consisting of 20 items rated on a 4-point scale. Similar to the anxiety scale, a standard score exceeding 53 indicates the presence of depression symptoms. Notably, higher scores indicate more severe depression symptoms; Cronbach α was 0.85.^[2]^

#### 2.2.5. Stigma Scale:

Utilized the Chinese version of the stigma scale developed by Deng Cuiyu,^[15]^ including 2 dimensions - internal and external stigma - with a total of 24 items rated on a 5-point scale. The total score ranges from 24 to 120, with higher scores indicating greater stigma. Cronbach α was 0.951.

### 2.3. Survey Method

This study was approved by the Ethics Committee of the First Hospital of Hebei Medical University (Ethics Number: 20220560). All participants provided written informed consent(The Declaration of Helsinki for completeness). Before data collection, a single designated investigator explained the study’s purpose and confidentiality policy to each patient and demonstrated how to complete the questionnaire; patients then completed the forms themselves. After the questionnaires were collected, they were checked, and any incomplete or inadequate questionnaires were excluded. A total of 129 questionnaires were distributed, with 5 being deemed ineligible, resulting in a response rate of 96.12%. Furthermore, a total of 124 valid questionnaires were collected; patients were sequentially assigned to the derivation and validation sets in a 3:1 ratio according to their clinic visit order.

### 2.4. Data Processing

The data were verified by 2 individuals and entered into the SPSS 26.0 software for statistical analysis. Normally distributed continuous variables were compared with the t-test. Non-normally distributed continuous variables with the nonparametric rank-sum test, and categorical variables with the χ^2^ test. In this study, variables with *P* < .05 in univariable analysis were entered into the logistic regression model. Based on the results of the logistic regression analysis, a stigma risk prediction scoring table was constructed.^[16]^ Furthermore, the model was internally validated on a validation set, and its effectiveness was evaluated by plotting the receiver operating characteristic (ROC) curve and calculating the area under the curve (AUC). The model calibration was then assessed using a calibration curve.

## 3. Results

Based on the stigma scores of the 93 patients in the derivation cohort, the median value was 35. The derivation cohort was therefore split into a high-score group (n = 47) and a low-score group (n = 46) using this median cutoff; the same cut-point was applied to classify the 31 patients in the validation cohort. The median score^[17]^for stigma among the included patients was 35 points. Based on the median score, the overall sample was divided into a high-score group (n = 47) and a low-score group (n = 46), with a total of 93 cases used for modeling, 31 cases used for modeling model validation.

### 3.1. Univariate analysis

The univariate analysis showed that, compared with the low-score group, the high-score group had a significantly higher proportion of patients with fertility desire (63.8% vs 39.1%, *P* < .05), hirsutism (83.0 % vs 30.4%, *P* < .01), acne (80.9% vs 32.6%, *P* < .01) and acanthosis nigricans (78.7% vs 37.0%, *P* < .01). Mean anxiety scores were also higher in the high-score group (50.06 ± 6.98) than in the low-score group (40.15 ± 5.64, *P* < .01). In contrast, mean depression scores were lower in the low-score group (47.13 ± 9.08) than in the high-score group (57.30 ± 7.77, *P* < .01). shown in Table 1.

**Table 1:** Single-factor analysis of stigma.

Aspect	Low-score group (n = 46)	High-score group (n = 47)	X^2^/t/z	*P*
Age(x ± s, years)	27.32 ± 5.718	28.87 ± 5.987	−1.714	.087
Education (n [%])			6.884	.076
Primary School	2 (4.3%)	4 (8.5%)		
Secondary School/Technical School	4 (8.7%)	3 (6.4%)		
College	16 (34.8%)	27 (57.4%)		
Bachelor degree and above	24 (52.2%)	13 (27.7%)		
Body mass index	29.27 ± 2.872	30.32 ± 4.564	-0.692	.489
Hirsutism (n [%])			26.185	**.000**
Not present	32 (69.6%)	8 (17.0%)		
Present	14 (30.4%)	39 (83.0%)		
Acne (n [%])			22.073	**.000**
Not present	31 (67.4%)	9 (19.1%)		
Present	15 (32.6%)	38 (80.9%)		
Acanthosis Nigricans (n [%])			16.655	**.000**
Not present	29 (63.0%)	10 (21.3%)		
Present	17 (37.0%)	37 (78.7%)		
Fertility requirements (n [%])			5.679	.017
No fertility requirements	28 (60.9%)	17 (36.2%)		
Fertility requirements	18 (39.14%)	30 (63.8%)		
Anxiety (x ± s, score)	40.15 ± 5.637	50.06 ± 6.976	7.527	**<.0001**
Depression (x ± s, score)	47.13 ± 9.079	57.30 ± 7.771	5.806	**<.0001**

t denotes continuous variables and χ^2^ denotes categorical variables.

*P* < .05 was considered statistically significant. *P* < .01 was considered highly significant.

### 3.2. Logistic regression analysis

Variables that were statistically significant in the univariate analysis were included in the logistic regression analysis. The results revealed that fertility requirements (OR=0.212, 95%Cl: 0.051–0.890, *P* < .05) , hirsutism (OR=0.075, 95%Cl: 0.015–0.368, *P* < .01) , acne (OR=0.210,95%Cl: 0.050–0.878, *P* < .05) , acanthosis nigricans (OR=0.184, 95%Cl: 0.044–0.073, *P* < .05) , and anxiety (OR=1.217, 95%Cl: 1.074–1.378,*P* < .01) were influencing factors for stigma in overweight and obese patients with PCOS (*P* < .05), as shown in Table 2.

**Table 2:** Logistic regression analysis results.

Factors		Prediction model			
	Regression coefficients	Standard error	WaldX^2^	Odds ratio (95% confidence Interval)	*P*-value
Constant	−5.789	2.912	3.951	0.003 (-)	.003
Hirsutism	−2.594	0.814	10.167	0.075 (0.015–0.368)	.001
Acne	−1.559	0.729	4.572	0.210 (0.050–0.878)	.032
Acanthosis Nigricans	−1.693	0.733	5.34	0.184 (0.044–0.073)	.021
Fertility requirements	−1.551	0.732	4.492	0.212 (0.051–0.890)	.034
Anxiety	0.196	0.064	9.494	1.217 (1.074–1.378)	.002

*P* < .05 was considered statistically significant. *P* < .01 was considered highly significant.

### 3.3. Construction of personalized prediction model

**Figure 1.**
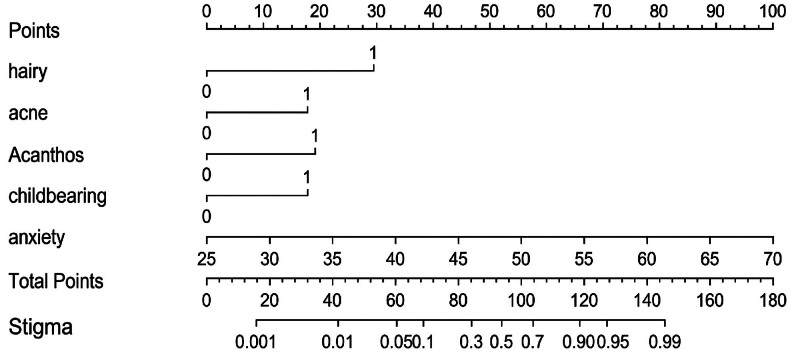
Nomogram of stigma prediction model for overweight and obese polycystic ovary syndrome patients.

**Figure 2.**
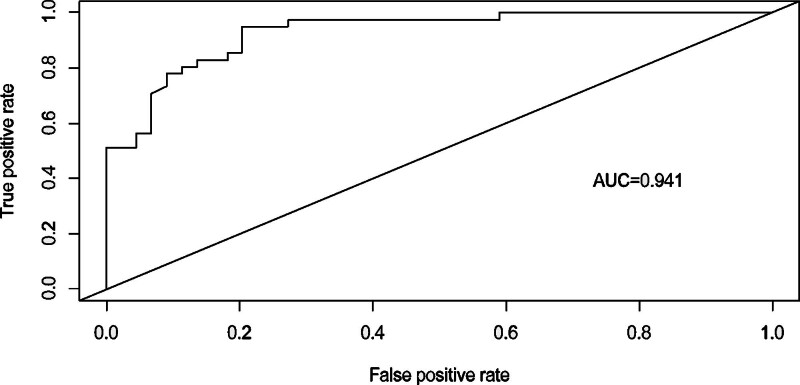
ROC curve of stigma model for overweight and obese patients with polycystic ovary syndrome. AUC = 0.941, indicating excellent discriminative ability.

**Figure 3.**
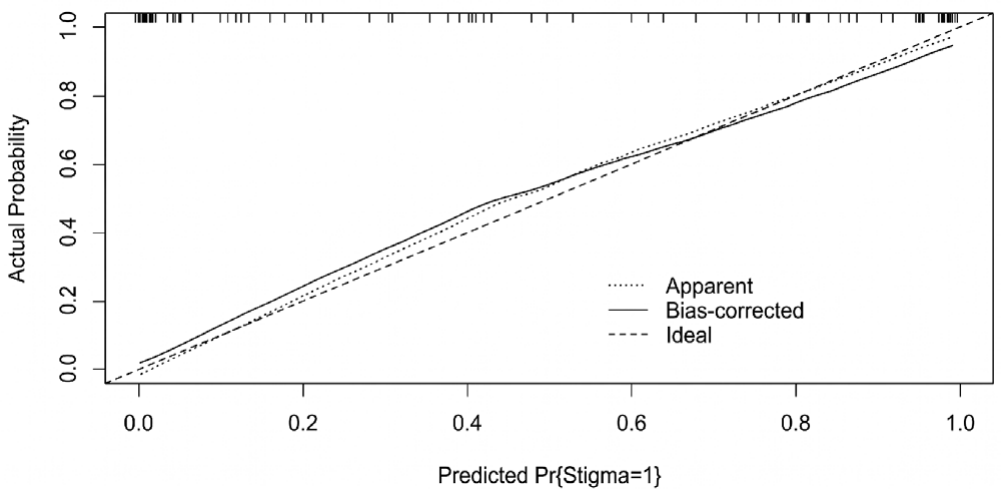
Calibration curve of stigma model for overweight and obese patients with polycystic ovary syndrome. Mean absolute error = 0.033. In the calibration plot, the curves of predicted probabilities and observed event rates are extremely close to the ideal reference line, indicating good calibration of the prediction model.

A column table framework was constructed in R using the 5 measurement factors identified in the logistic regression analysis (Fig. 1). The ROC curve was plotted with an AUC of 0.941 (95% CI: 0.896–0.985, *P* < .001),the model exhibited a sensitivity of 0.957, a specificity of 0.804, and a Youden index of 0.761, indicating excellent discriminative ability set (Fig. 2). The calibration curve was then used to evaluate the stigma prediction model. In the calibration plot, the mean absolute error was 0.033. The curves of predicted probabilities and observed event rates are extremely close to the ideal reference line, indicating good calibration of the prediction model (Fig. 3).

### 3.4. Model performance and internal validation

**Figure 4.**
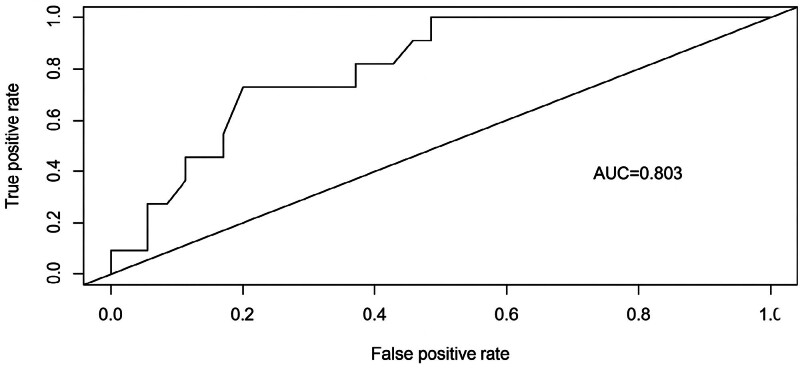
ROC curve of stigma validation for overweight and obese patients with polycystic ovary syndrome. AUC = 0.803, indicating good discriminative ability.

**Figure 5.**
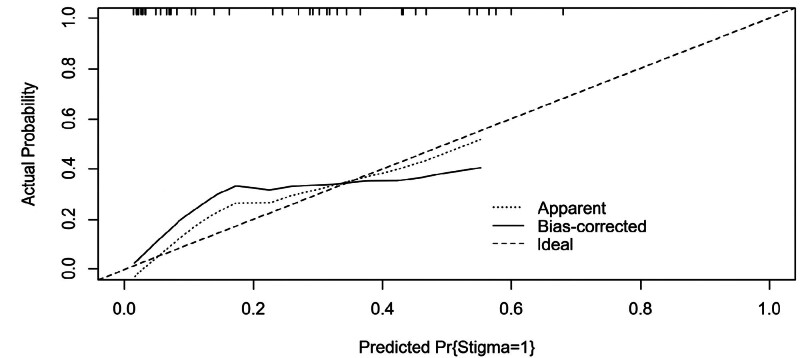
Calibration curve of stigma validation for overweight and obese patients with polycystic ovary syndrome. The calibration curve yielded a mean absolute error of 0.07, indicating good agreement between the model-predicted probabilities and the actual risk of stigma in patients, thus confirming reliable internal validity.

**Figure 6.**
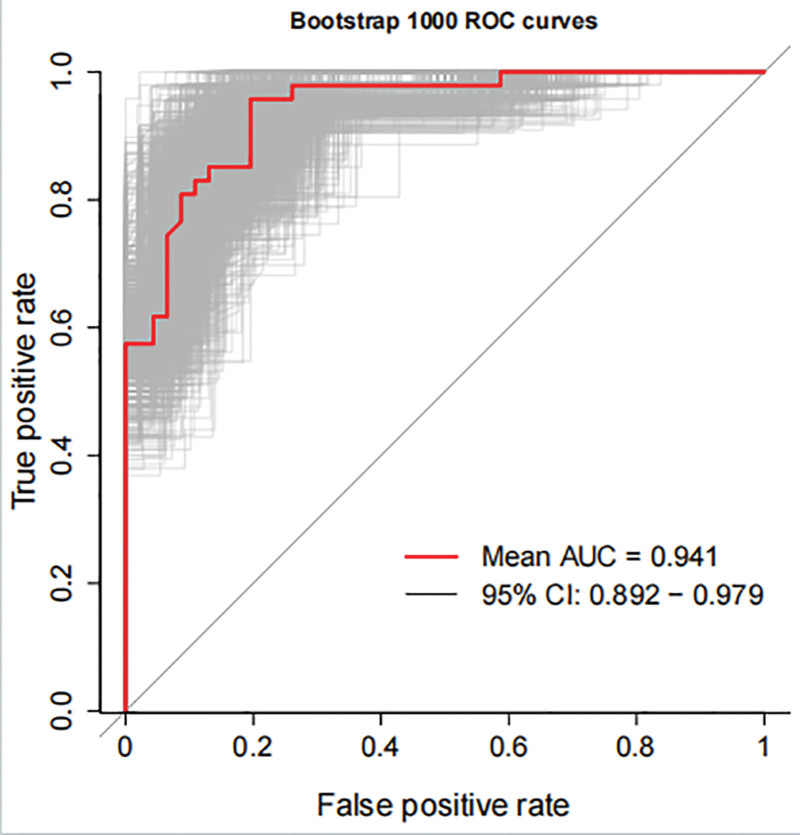
Bootstrap 1000 ROC curves. The mean AUC from bootstrap validation was 0.941 (95% CI: 0.892–0.979), confirming the robust performance of the model and mitigating the risk of overfitting.

Model validation was conducted using 31 patients. The ROC curve was plotted, with an AUC of 0.803 (95% CI: 0.675–0.921, *P* < .001),yielding a sensitivity of 0.727, a specificity of 0.829, and a Youden index of 0.556, indicating good discriminative ability in identifying stigma among PCOS patients(Fig. 4). Additionally, the calibration curve yielded a mean absolute error of 0.07, indicating good agreement between the model-predicted probabilities and the actual risk of stigma in patients, thus confirming reliable internal validity(Fig. 5).To further validate the stability and generalizability of the model, internal validation was conducted using the bootstrap method with 1000 resamples. The mean AUC from bootstrap validation was 0.941 (95% CI: 0.892–0.979), confirming the robust performance of the model and mitigating the risk of overfitting (Fig. 6).

## 4. Discussion

This study conducted univariate analysis, logistic regression analysis, and model establishment based on general information, physical signs, anxiety, and depression of overweight and obese patients with PCOS. Hirsutism, acne, acanthosis nigricans, fertility-desire status, and anxiety were identified as independent determinants of stigma in these patients. Ultimately, A predictive model was then developed based on these 5 independent factors. This model was validated, and we demonstrated its effectiveness in predicting stigma in patients with this condition. Furthermore, this provides a basis for the early identification of overweight and obese patients with PCOS with high levels of stigma in clinical practice, enabling interventions and enhancing patients’ quality of life.

Patients with PCOS often exhibit symptoms of hyperandrogenism and insulin resistance, Acanthosis nigricans is commonly observed on the skin in areas such as the groin, neck, and armpits, presenting as gray-brown discoloration.^[18]^ Unfortunately, some patients may experience issues in one aspect, while others may have multiple concerns simultaneously, significantly impacting their appearance. Moreover, excessive hair growth is typically prominent on the upper lip, inner thighs, and lower abdomen.^[19]^ Influenced by traditional Chinese cultural standards that emphasize smooth, fair skin, women often view excessive hair growth as aesthetically undesirable. With the rapid development of the medical aesthetics industry, technologies such as laser treatments, waxing, and bleaching can help reduce the impact of facial hair on appearance.^[20]^ However, excess hair growth still affects an individual’s work and daily life, leading to reduced quality of life.^[21]^ Additionally, acne commonly occurs on the chest, back, shoulders, and face, with facial acne prevalent, and hirsutism can affect satisfaction with self-image and social interactions.^[22]^ These symptoms can lead patients to feel “unattractive” and to fixate on their perceived flaws. In social settings, they worry about being judged, so they avoid gatherings to escape stares and comments; in romantic contexts, they fear a partner’s negative appraisal, which may prevent them from starting a relationship or cause embarrassment during intimacy; at work, especially in public-facing roles, the persistent sense of “not being beautiful” erodes self-confidence and impairs performance. Consequently, these result in anxiety, stigma, and other psychological issues.^[23]^ Thus, the findings of this study align with these observations.

This study indicates that patients with fertility requirements exhibit higher levels of stigma compared to those without fertility requirements. Children are the fruit of love; many couples long to witness a new life grow, to taste the joy of parenthood, to let their love continue, and to enrich family life. Due to the influence of hyperandrogenism, patients with PCOS may experience anovulation or irregular ovulation, with PCOS being the most common cause of anovulatory infertility.^[12]^ Moreover, as women with PCOS reach childbearing age, seeing friends and colleagues having their children and pressure from both sides of the family can exacerbate the psychological burden on patients with PCOS.^[24]^ Studies have also demonstrated that anxiety, depression, and other emotional issues are common among women with infertility, and there is a reciprocal relationship between infertility and emotional well-being.^[25]^ Thus, negative psychological experiences may lead to a lack of confidence in patients. Therefore, this study demonstrates a correlation between anxiety and stigma, consistent with previous research findings.^[26]^

To address the risk factors of stigma in overweight and obese patients with PCOS, the following intervention strategies can be applied:

1. Establish a clinical pathway to screen at-risk patients during diagnosis and treatment, and implement a multidisciplinary management model involving endocrinology, psychology, dermatology, and other relevant departments to provide comprehensive disease management.
2. Healthcare professionals can provide personalized disease education to patients, allowing them to have a better understanding of their condition, be informed about the main treatment options, and effectively learn about management strategies.
3. Encourage patients to express their concerns and needs to their family members, enabling patients to receive care and support from loved ones, thereby alleviating feelings of guilt and stigma.
4. Respect patients fully, protect their privacy, and refrain from disclosing their medical condition to others without the patient’s consent.

## 5. Limitations

First, the sample size of this study is limited, and further validation is needed by expanding the sample. Second, only the factors related to stigma in overweight and obese PCOS patients were studied, while the factors related to stigma in PCOS patients with normal weight were not analyzed. Third, Median split of the stigma score and dichotomizing a continuous outcome may reduce statistical power, the scale has not been validated in PCOS populations, limiting generalizability. Future studies could use continuous variables and validate the scale in PCOS patients. Additionally, as this study employed convenience sampling, the findings may be subject to selection bias; formal clinical implementation should therefore be preceded by external validation.

## 6. Conclusion

This model demonstrated strong predictive performance and may assist clinicians in identifying women at risk of stigma, facilitating early psychological and educational interventions. However, due to the absence of external validation, the clinical application should be viewed with caution. Widespread clinical implementation is not currently supported, and further external validation is needed before clinical translation.Future studies might include larger, multicentre models across different populations.

## Acknowledgments

The authors gratefully acknowledge the time and effort of the participants involved in our study. The authors thank AiMi Academic Services (www.aimieditor.com) for English language editing and review services.

## Author contributions

**Data curation:** Yahui Bian.

**Formal analysis:** Zijie Fu, Tingting Zhao.

**Funding acquisition:** Zijie Fu, Tingting Zhao.

**Investigation:** Tingting Zhao, Jingjing Wang.

**Methodology:** Yahui Bian.

**Project administration:** Zijie Fu, Xiaodong Li.

**Resources:** Yanan Bian, Bianling Xu.

**Software:** Yanan Bian, Bianling Xu.

**Supervision:** Yahui Bian.

**Writing – original draft:** Jingjing Wang.

**Writing – review & editing:** Qingya Ma.
